# Supplementary material for: Distinct Subtyping of Successful Weaning from Acute Kidney Injury Requiring Renal Replacement Therapy by Consensus Clustering in Critically Ill Patients
Source: Biomedicines. 2022 Jul 7;10(7):1628. doi: 10.3390/biomedicines10071628 (PMC9313082; doi:10.3390/biomedicines10071628)
Supplement: Supplementary file 1 [file biomedicines-10-01628-s001.zip › biomedicines-1758916-supplementary.pdf]

Supplemental Digital Content

This Supplemental Digital Content contains the following material:

Study Design
Kit Detection Ranges
Table S1. Reason of re-dialysis
Table S2. NRI and IDI analyses for the role of uNGAL/Cr in stratifying individuals into high or low risk of mortality
Figure S1. Decision curve analysis (DCA) plot to assess the clinical consequences of screening patients for the risk of 90-day mortality using uNGAL/Cr
Figure S2. Graphic abstract

Study design

Time course of the study parameters:

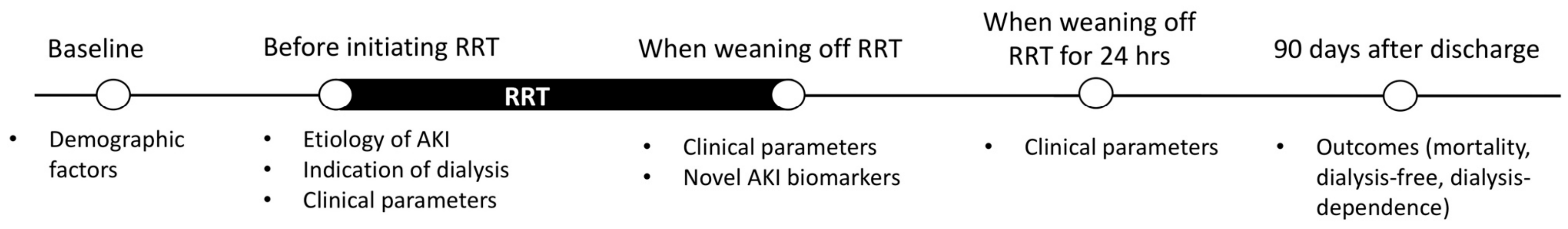

Abbreviations: AKI, acute kidney injury; RRT, renal replacement therapy

Kit Detection Ranges. L-FABP, NGAL enzyme-linked immunosorbent assay (ELISA) kits.

The concentrations of novel AKI biomarkers were determined using enzyme-linked immunosorbent assay (ELISA) kits [Renal liver-type fatty acid binding protein (L-FABP), Sekisui Medical Co., Ltd., Tokyo, Japan; Neutrophil gelatinase associated lipocalin (NGAL), R&D Systems, Minnesota, USA] when weaning off RRT. Each biomarker assay was performed in duplicate according to the manufacturer's instructions, and the mean value was used for further statistical analysis. The associated information of the commercial ELISA kits are listed below:

Intra- and inter-assay coefficients of variation:
L-FABP: < 3% and < 15%, respectively.
NGAL: < 5% and < 8%, respectively.

Detection limits:
L-FABP: 4 ng/mL
NGAL: 0.04 ng/mL

Reference intervals:
L-FABP: 1.0 - 200 ng/mL
NGAL: 0.2 - 10 ng/mL

Abbreviations: AKI, acute kidney injury; ELISA, enzyme-linked immunosorbent assay; L-FABP, liver-type fatty acid binding protein; NGAL, neutrophil gelatinase-associated lipocalin; RRT, renal replacement therapy.

Table S1. Reason of re-dialysis

**Predictors¶**	**Total (n=124)**	**Cluster1 (n=30)**	**Cluster2 (n=16)**	**Cluster3 (n=78)**	**P value**
Azotemia, n (%)	33 (26.6%)	9 (30.0%)	3 (18.8%)	21 (26.9%)	0.45
Fluid overload, n (%)	6 (4.8%)	0 (0.0%)	1 (6.3%)	5 (6.4%)	0.99
Electrolyte imbalance, n (%)	1 (0.8%)	0 (0.0%)	0 (0.0%)	1 (1.3%)	0.31
Acid-base imbalance, n (%)	3 (2.4%)	1 (3.3%)	1 (6.3%)	1 (1.3%)	0.71
Uremic symptom, n (%)	3 (2.4%)	1 (3.3%)	1 (6.3%)	1 (1.3%)	**0.001**
Oliguria/anuria, n (%)	15 (12.1%)	8 (26.7%)	1 (6.3%)	6 (7.7%)	**0.028**

¶ Criteria of re-dialysis: If renal impairment had progressed to one or more of the following: (1) azotemia (blood urea nitrogen > 80 mg/dL and serum creatinine > 2 mg/dL) with uremic symptoms (encephalopathy, pericarditis, or pleuritis); (2) hyperkalemia (serum potassium level > 5.5 mmol/L) refractory to medical treatment; (3) oliguria (urine output < 400 mL/24h) or anuria refractory to diuretics; (4) fluid overload refractory to diuretics with a central venous pressure > 12 mmHg or pulmonary edema with PaO_2/FiO2 < 300 mmHg; and (5) metabolic acidosis (pH < 7.2 in arterial blood)

Table S2. NRI and IDI analyses depicting the role of uNGAL/Cr in stratifying individuals into high or low risk of 90-day mortality.

Model	Model with uNGAL/Cr (log)		
Freq (Row percent)	< 30%	≥ 30%	Total(no.)/reclassification(%)
Patients who died			
<30%	35	15	50/30
>=30%	19	17	36/53
Total	54	32	86
Patients who survived			
<30%	3	15	18/83
>=30%	4	16	20/20
Total	7	31	38
Combined data			
<30%	38	30	68/44
>=30%	23	33	56/41
Total	61	63	124

NRI(Categorical) [95% CI]: 0.336 [0.092 - 0.580]; *P*-value: 0.007

NRI(Continuous) [95% CI]: 0.700 [0.348 - 1.053]; *P*-value: < 0.001

IDI [95% CI]: 0.090 [0.041 - 0.138]; *P*-value: < 0.001

IDI for events [95% CI]: 0.289 [0.084 - 0.495]; *P*-value: 0.006
IDI for non-events [95% CI]: 0.047 [-0.086 - 0.179]; *P*-value: 0.49

Abbreviations: CI, confidence interval; Cr, creatinine; IDI, integrated discrimination improvement; NGAL, neutrophil gelatinase-associated lipocalin; NRI, net reclassification improvement.

Figure S1. Decision curve analysis plot to assess the clinical consequences of screening patients for the risk of 90-day mortality using uNGAL/Cr. Y-axis is the net benefit of the decision strategy. Net benefit was defined as the net proportion of patients with 90-day mortality in whom a prediction model would provide benefit without applying a prediction model to patients with good outcomes. For the survivors (black line), forecasting with an adding of uNGAL/Cr did not yield a net benefit. When considering those who died (grey), clear net benefits were seen for risk thresholds from 30 to 40%, forecasting that an adding of uNGAL/Cr was beneficial (blue line). The net benefit was calculated as (proportion of true positives) – (proportion of false positives)*pt/(1-pt), where pt is the threshold probability.

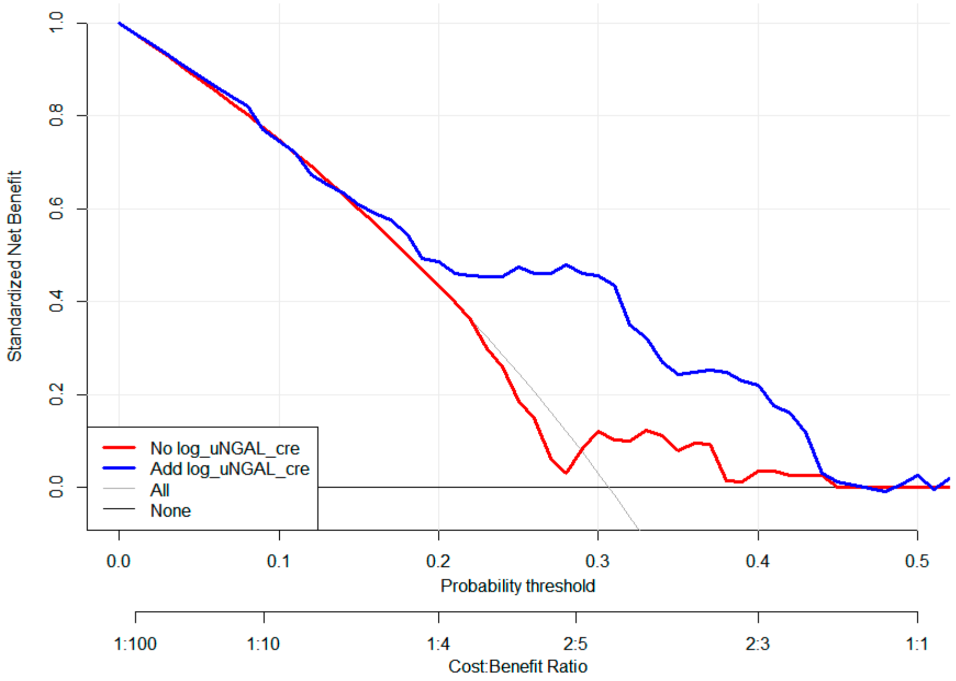

Abbreviations: Cr, creatinine; NGAL, neutrophil gelatinase-associated lipocalin.
